# Model-guided geospatial surveillance system for antimalarial drug resistance

**DOI:** 10.1371/journal.pgph.0004717

**Published:** 2026-01-06

**Authors:** Apoorv Gupta, Lucinda E. Harrison, Minu Nain, Sauman Singh Phulgenda, Rutuja Chhajed, Roopal S. Kumar, Aishika Das, Manju Rahi, Philippe J. Guerin, Anup R. Anvikar, Mehul Dhorda, Jennifer A. Flegg, Praveen K. Bharti

**Affiliations:** 1 ICMR-National Institute of Malaria Research, New Delhi, India; 2 Infectious Diseases Data Observatory (IDDO), Oxford, United Kingdom; 3 School of Mathematics and Statistics, University of Melbourne, Parkville, Victoria, Australia; 4 WorldWide Antimalarial Resistance Network (WWARN), Oxford, United Kingdom; 5 Centre for Tropical Medicine and Global Health, Nuffield Department of Medicine, University of Oxford, Oxford, United Kingdom; 6 ICMR-Vector Control Research Centre (VCRC), Medical Complex, Puducherry, India; 7 Academy of Scientific and Innovative Research (AcSIR), Ghaziabad, Uttar Pradesh, India; 8 Mahidol Oxford Tropical Medicine Research Unit, Faculty of Tropical Medicine, Mahidol University, Bangkok, Thailand; PLOS: Public Library of Science, UNITED STATES OF AMERICA

## Abstract

Disease surveillance activities are usually resource-constrained and should be optimised to generate the most informative scientific findings, and to make the best use of time, finances, and personnel. India has a high population density, diverse geography and climatic conditions, and difficult terrain. With respect to malaria, *Plasmodium falciparum* and *Plasmodium vivax* are endemic, with substantial variability of transmission across the country. While for *P. vivax*, drug efficacy appears to be homogeneous within the country, for *P. falciparum* malaria, the drug resistance pattern varies from the northeastern region to the central region. Accounting for these complexities, we develop a decision-making framework guided by geospatial modelling outputs to identify prospective study sites for surveillance of molecular markers of antimalarial drug resistance in *P. falciparum* malaria in India. We first retrieve existing data on the prevalence of validated markers of resistance to artesunate and sulfadoxine-pyrimethamine from the World Wide Antimalarial Resistance Network (WWARN) Surveyor database. We then incorporate these data into a geostatistical model to estimate the prevalence of these markers across India and identify areas with high median estimated marker prevalence and high uncertainty. Finally, we create an interactive dashboard using the RShiny software package to simplify the process of selecting sites for future molecular surveillance. Our framework helps to ensure that operational decision-making is supported by data and modelling outputs. We demonstrate the utility of our framework by selecting sites for molecular surveillance of *P. falciparum* malaria in India.

## Introduction

Malaria remains a significant public health problem despite global efforts to control and eliminate the disease [1]. The World Health Organization (WHO) in its Global Technical Strategy (GTS), adopted in May 2015, set the goal of a 90% reduction in malaria cases worldwide by 2030 [2]. Several medicines are currently used for the treatment of uncomplicated *Plasmodium falciparum* malaria. They are all based on artemisinin combination therapy (ACT), a treatment combining an artemisinin derivative with a partner drug, e.g., lumefantrine, amodiaquine, pyronaridine, mefloquine, piperaquine or sulfadoxine-pyrimethamine. In mid-2000, artemisinin resistance was first reported in Cambodia, followed by further spread or emergence in other parts of the Greater Mekong Subregion [3,4]. In mid-2010, artemisinin resistance was identified in Rwanda and Uganda and has since been confirmed or suspected in eight countries in sub-Saharan Africa [5]. Without a feasible non-artemisinin-based treatment for *P. falciparum* infections in the near future, the geographic expansion of artemisinin resistance poses a significant threat to malaria control in all endemic areas. In addition, the dramatic decrease in available funding to fight malaria worldwide, following the dismantling of the President’s Malaria Initiative, raises further concerns about the capacity to maintain the fragile gains obtained since the beginning of the millennium.

To mitigate the risk, real-time surveillance of antimalarial drug resistance is key to gathering evidence to guide policies and propose alternative strategies, such as changes of ACT in first-line therapy, use of gametocidal drugs, and enhanced vector control measures [5–8]. Drug resistance surveillance aims to identify, monitor, and map regions where drug-resistant malaria strains are prevalent, through the collection and analysis of data on malaria transmission, drug efficacy, and patient outcomes [1,4]. *In vivo* therapeutic efficacy studies generate direct estimates of treatment failure rates in the population, but are relatively costly and time-consuming to conduct [9]. These studies are complemented by the collection of parasite sequencing data, which documents population-level prevalences of genetic markers for drug resistance. While the prevalence of genetic markers associated with delayed parasite clearance may be indirect evidence of rising treatment failure, molecular surveillance studies can include greater numbers of participants for significantly less financial investment and time commitment per participant [9], thereby informing public health policies and interventions.

All forms of antimalarial drug resistance surveillance are resource-intensive and time-consuming. Available resources must be utilised optimally to improve surveillance coverage and achieve close to real-time situational awareness. mathematical models can provide an additional evidence base for disease surveillance decision-making as they allow us to predict and visualise disease transmission patterns, treatment efficacy, and the impact of intervention strategies [10,11]. Geospatial models can characterise the spatiotemporal distribution of malaria transmission and identify areas for targeted intervention [10]. Geospatial modelling has been used to support antimalarial resistance surveillance efforts by identifying areas of highest value to surveillance, using both predicted prevalence of molecular markers for drug resistance and uncertainty in model predictions [12,13].

Following the recommendation of the WHO GTS, India launched its ‘National Framework for Malaria Elimination’ in 2016. Under this framework, India aims to eliminate malaria, prevent reintroduction, and maintain malaria-free status across the country by 2030 (Ministry of Health and Family Welfare, 2016). Mutations in the propeller region of the *pfkelch13* gene have been found to be associated with resistance to Artemesnin [14]. These mutations were first identified in the Greater Mekong Sub region, but have since been reported in north eastern India and central and eastern parts of sub-Saharan Africa [8]. With the increase of *Plasmodium falciparum* (Pf) malaria cases in India (NCVBDC, 2024) [15], the rise of SP resistance [16,17], and the identification of *pfkelch13* mutations in the northeastern region [17], synthesis of accumulated evidence of antimalarial resistance, through review, mapping, and modelling, is a priority to achieve the goals of the National Framework for Malaria Elimination by 2030 [16,17].

India has complex geographical boundaries, varying temperatures and climates, and vast river basins. Pf and *Plasmodium vivax* (Pv) are endemic across the country, with large heterogeneity in prevalence and transmission. The National Centre for Vector Borne Diseases Control reported approximately 130,000 laboratory-confirmed Pf malaria diagnoses in 2024 (NCVBDC, 2024), although this is likely an under-estimate of the number of cases [6,15]. Different regions in India require different Pf malaria drug interventions, adding to the complexity of surveillance efforts (Ministry of Health and Family Welfare, 2016). The current recommended treatment for uncomplicated Pf malaria is artemether-lumefantrine (AL) in the northeast region of India since 2013 and artesunate-sulphadoxine-pyrimethamine (AS+SP) in the rest of India, because of the occurrence of SP resistance in the northeast [18,19]. To optimise current surveillance efforts, a targeted framework is needed to identify prospective study sites where estimates of parasite susceptibility to antimalarials, via molecular surveillance data, are most urgently needed. The use of geospatial modelling tools and dynamic dashboards can aid surveillance efforts by identifying priority areas based on specific decision objectives and constraints.

We develop a framework for model-guided site selection for the surveillance of molecular markers of antimalarial drug resistance, using tailor-made geospatial models for India. The models use existing data on the prevalence of genetic markers of antimalarial drug resistance in India, including known mutations in *P. falciparum* dihydropteroate synthase (*pfdhps*) and dihydrofolate reductase (*pfdhfr*), which are markers for sulfadoxine-pyrimethamine (SP) resistance, and validated markers in the *kelch13* region (*pfk13*)*,* which are markers for artemisinin resistance [20]*.* We use publicly available data to inform geostatistical models of resistance marker prevalence, which provide both a surface of predicted marker prevalence for India and a surface of prediction uncertainty. To facilitate site selection, we create a dashboard using the RShiny software package [21]. The districts selected using the RShiny dashboard are presented in visual and tabular formats with the districts ranked based on a set of epidemiological parameters. The dashboard facilitates data-driven decision-making and visualises the current status of prevalence of molecular markers for antimalarial drug resistance in India. Our methodology is piloted to identify sites for future molecular surveillance.

## Methodology

### Data extraction from WWARN DHPS and K13 systematic review database

In this study, we used data on the prevalence of *pfdhfr/pfdhps* and *pfk13* mutations across India. The data was obtained from databases of *pfdhps/pfdhfr* and *pfk13* resistance markers accessible on the WWARN Molecular Surveyors (https://www.iddo.org/wwarn/tracking-resistance). The WWARN Surveyors constitute a living systematic review of published and unpublished studies reporting population-level prevalence of molecular markers of *P. falciparum* resistance to SP (*pfdhps*, *pfdhfr*) and to artemisinin (*pfk13*). In addition, we included data recently identified in the systematic review of molecular surveillance of *P. falciparum* in India conducted by Nain et al. [22]. This review contributed nine published studies and one unpublished study to the final dataset. Data were extracted only from those studies in the WWARN database that reported complete information on the location and period of sample collection, and the prevalence of the markers of interest. Extracted data contain study title, author details, study year (precise or estimated), publication year, geographic location (precise or estimated), number of successfully sequenced samples for each study site, and number of samples with wild-type parasites or with WHO-validated single nucleotide polymorphisms (SNPs) associated with SP resistance (*pfdhps*/*pfdhfr*) or artemisinin resistance (*pfk13*). Strains carrying the *pfdhps540E* mutation are uncommon, and the mutation is typically seen in conjunction with additional *pfdhps* and *pfdhfr* mutations in the form of quintuple and sextuple mutations. As a result, *pfdhps540E* is commonly used as a proxy for quintuple and sextuple *pfdhps*/*pfdhfr* mutations linked with clinical SP resistance [23–25].

### Spatiotemporal statistical modelling

The *pfdhps540E* and *pfk13* datasets were used in a Bayesian geostatistical model to produce predictive maps of the prevalence of the markers in India. The statistical methodology for spatiotemporal prediction has been documented previously and follows two stages (see S1 Text) [10,12,25]. In the first stage, the posterior distribution of model parameters given the observed data was estimated. Convergence diagnostics for the Bayesian inference were monitored, including trace plots, autocorrelation, and coverage probabilities; no evidence of divergence was found. In the second stage, the posterior predictive distributions of marker prevalences were predicted on a nominal 5 x 5 km^2^ resolution grid over India, given the posteriors for the model parameters from the first stage. The posterior predictive distributions at each location were summarised using the median and standard deviation to create point estimates and uncertainty surfaces, respectively. The modelling framework is described in detail in the S1 Text. We consider modelling outputs relevant to 2021 in the remainder of the workflow, as this is the year for which other epidemiological data is available. We visualise the model outputs using Administrative India Boundary shape files up to district level with HQ-Scale 1:1M (having State Boundary and District Boundary) from the survey of India with product code OVSF/1M/7.

**Supplementary Material:** The S1 Text contains a detail about how we develop geospatial model, with similarities and difference from existing models.

### Epidemiological datasets employed

In this study, we consider three malaria-specific epidemiology variables for categorising states and their associated districts from the national Malaria Annual Report 2023 (NCVBDC, 2023) [26]

1. Total number of Blood Slide Examinations (BSE) - Total number of blood slides examined under a microscope in a month to detect malaria parasite infection.
2. *Plasmodium falciparum* cases (Pf) - Total number of Pf malaria-positive cases per month with positive blood slide examination.
3. *Plasmodium falciparum Parasite Rate* (PFPR) - Total rate of Pf malaria positivity in a month:

$$PFPR=\frac{Pf}{BSE}\times \text{1}\text{0}\text{0}.$$

These parameters are integrated into the dashboard for ranking and clustering states and their associated districts into three groups of mild, moderate, and high Pf malaria burden.

### RShiny Dashboard

An RShiny dashboard was constructed to use modelling outputs and epidemiological data to facilitate the selection of districts for molecular surveillance [21]. As well as median posterior estimates of marker prevalence and posterior uncertainty, we include the additional contextual datasets of estimated gridded Pf parasite rate (PFPR) [27], estimated district accessibility [27], estimated human population density, and estimated temperature suitability for Pf transmission [28]. The dashboard allows the user to place a set of “filters” in a preferred order to exclude districts on the grounds they do not satisfy one of the filtering criteria. For example, a “filter” can be placed on the PFPR, to include only the 50 districts with the highest PFPR in further decision-making. The dashboard allows the results of multiple filtering outputs to be visualised and compared without the decision-maker being familiar with the R programming language or any of R’s functionality for geospatial modelling or visualisation. The workflow featured in our dashboard was iterated subject to project aims and data availability – the final workflow excludes districts based on filtering variables (i.e., a subtractive approach), rather than optimising one or more decision objectives (a more direct approach). The set of districts selected through the dashboard is highly sensitive to decisions made during the selection and ordering of filters, and the number of districts excluded as each filter is applied. This sensitivity is acknowledged, but allowable: the workflow presented is a quantitative, structured approach to decision-making and is preferable to selecting sites ignoring available data and without considering specific surveillance objectives (e.g., selecting equidistant sites or selecting sites at random) [29,30]. The dashboard is freely available at https://iddo.shinyapps.io/pf_drug_resistance.

### Workflow for site selection

**Fig 1:**
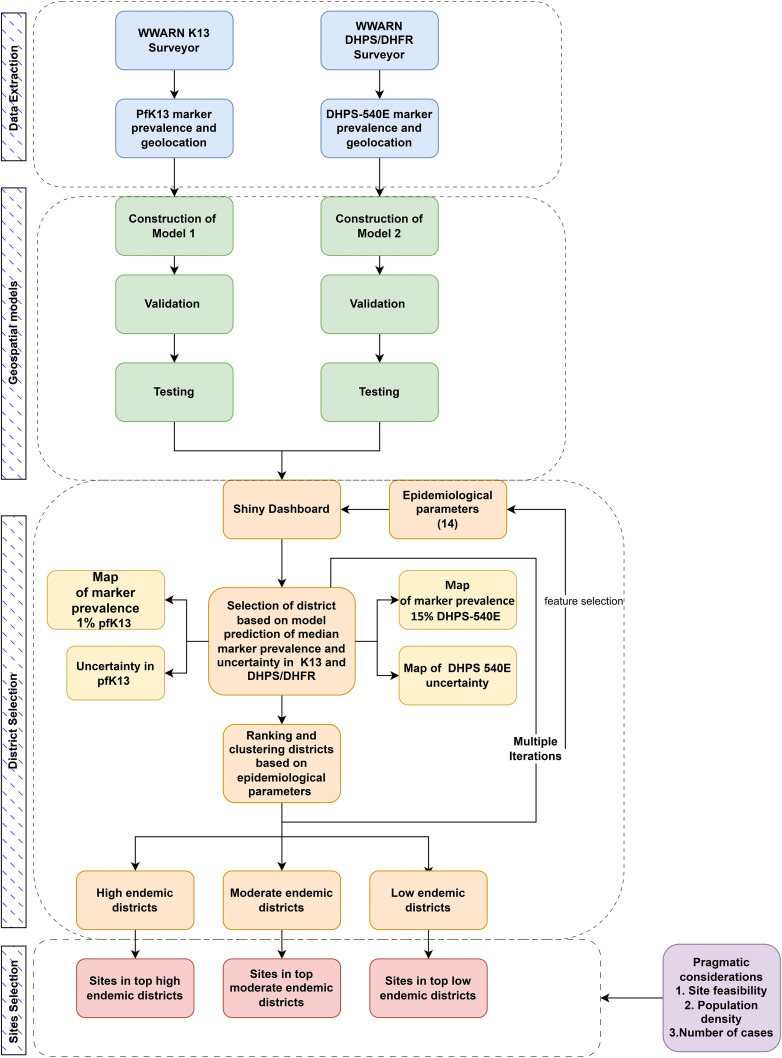
Flowchart describing the modelling and decision-making workflow: data are extracted to inform geospatial models; the outputs of geospatial models, together with other epidemiological parameters, inform district selection; surveillance sites are selected from filtered districts.

We selected four districts for surveillance, based on the geospatial modelling of markers for resistance and other epidemiological data across India (Fig 1). The RShiny dashboard integrates the two geospatial models with other epidemiological variables for filtering and ranking districts to identify sites for prospective surveillance. The workflow for district selection was agreed on after multiple iterations of possible decision objectives and constraints. The final workflow divided districts into three groups, by malaria endemicity (high, medium, and low). Pragmatic considerations including site feasibility, population density, and expected number of malaria cases that might be candidates for surveillance study inclusion, were used to finalise site selection from the filtered district-level outputs.

## Results

### Data summary

The extracted dataset from the WWARN Surveyors contains 113 *pfk13* and 67 *pfdhps*540E resistance marker records in India, reported between 1994 and 2019 in September 2022. We used the *pfdhps*540E geospatial model (with zero-time separation and a spatial correlation threshold of 1%) to calculate a reasonable cut-off distance of 675 km from the Indian border. This included studies that could affect the model from neighbouring countries; a total of 54 *pfk13* studies and 40 *pfdhps* studies having 155 *pfk13* data entries and 24 *pfdhps*540E entries, respectively, were extracted from six neighbouring countries, including Afghanistan, Pakistan, Nepal, Bangladesh, China, and Myanmar. Most of the Indian studies for *pfdhps*540E are from the East and Northeast regions. There are also 15 entries from the Central regions of India and 6 entries from the North for *pfdhps*540E. Most of the entries in the *pfk13 d*ataset are from the East and Northeast regions, 20 from the Central region, and a minority from the North (n = 4), South (n = 4), and West (n = 2) regions of India.

### Geostatistical model

Using the data, continuous predictive maps for *pfk13* and *pfdhps*540E from 2000 to 2022 were generated. The construction of the statistical model allows predictions to be made at locations in space and time where no data are available, and the models allow for quantification of uncertainty in predictions.

**Fig 2:**
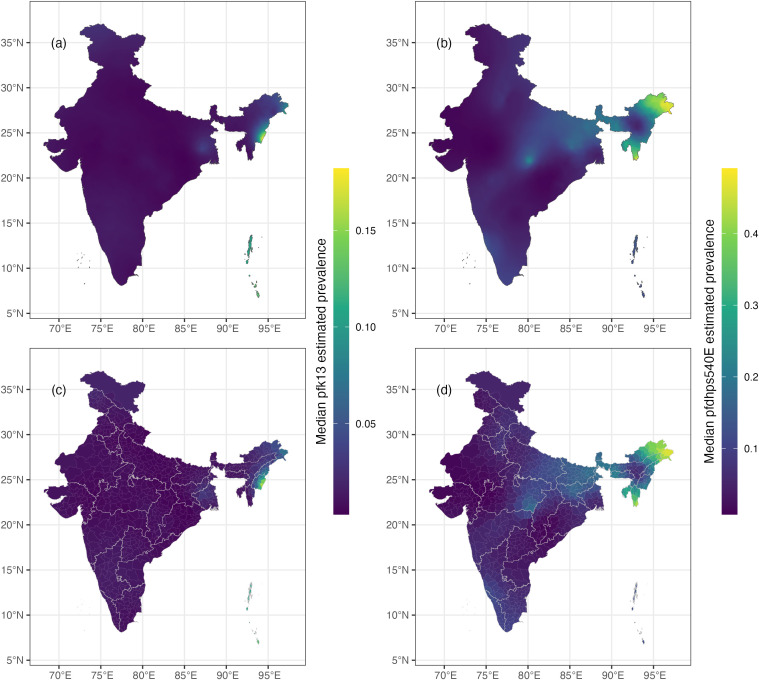
A. Median posterior predictive prevalence of *pfk13* in 2021; B median posterior predictive prevalence of *pfdhps540E* in 2021; subplots C and D show the median posterior predictive prevalence by district for *pfk13* and *pfdhps540E*, respectively.

**Fig 3:**
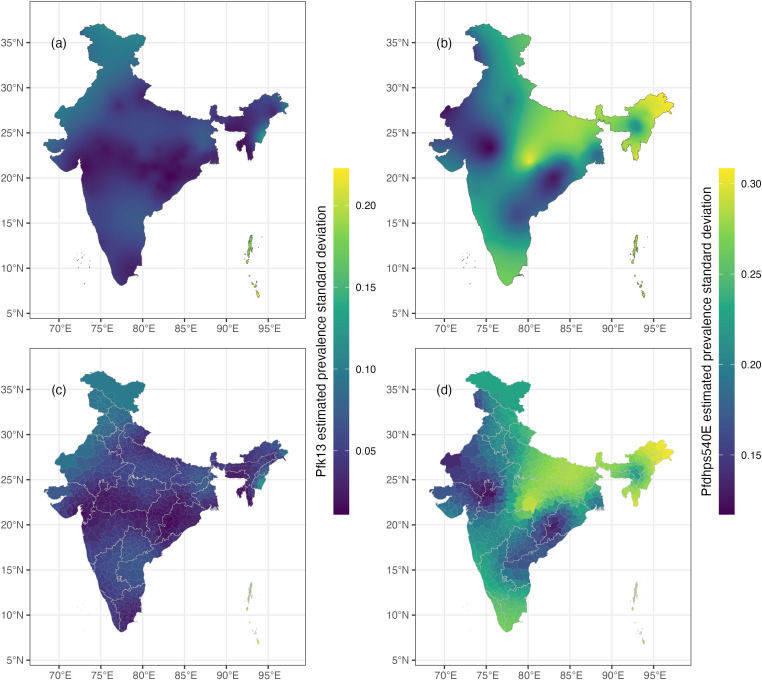
A. Standard deviation for *pfk13* posterior predictions in 2021, associated with median predictions in Fig 2A; Fig 3B standard deviation for *pfdhps540E* posterior predictions in 2021, associated with median predictions in Fig 2B. Subplots C and D show the standard deviation of posterior predictive prevalence by district for *pfk13* and *pfdhps540E*, respectively.

The median of the posterior predictive distribution of *pfk13* was near zero over the entire country in 2021, except for a few ‘hotspots’ in the Northeastern region (Fig 2A, Fig 2C). The associated uncertainty map (Fig 3A) showed relatively high certainty in these model results, but less certainty at the hotspots in the Northeast and Northwest regions. The median of the posterior predictive distribution of *pfdhps*540E was low across most of the country in 2021 but moderate in the Northeast, East, and Central regions (Fig 2B, Fig 2D). The associated uncertainty is high in regions of moderate median values (Fig 3B).

**Fig 4:**
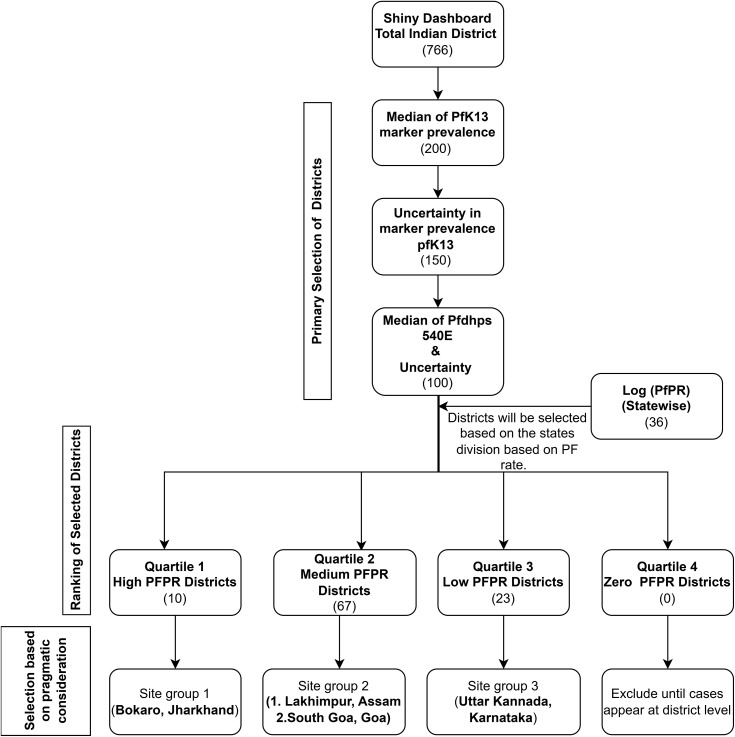
Flow diagram for district/site selection using the RShiny dashboard.

Out of 766 total districts across India, 200 were selected in the first step of the workflow as those with the highest median *pfk13* prevalence (Fig 4). Out of these 200 districts, 150 were selected as those having the highest standard deviation (SD) in *pfk13* prevalence, followed by the shortlisting of 100 districts with the highest median *pfdhps*. The latest 2022 state-level (PFPR) data was used to divide all states into the categories of high, medium, and low malaria burden – these categories were then assigned to the districts within each state. Of the shortlist of 100 districts, four districts were selected: one each from high- and low-burden states and two from medium-burden states.

**Fig 5:**
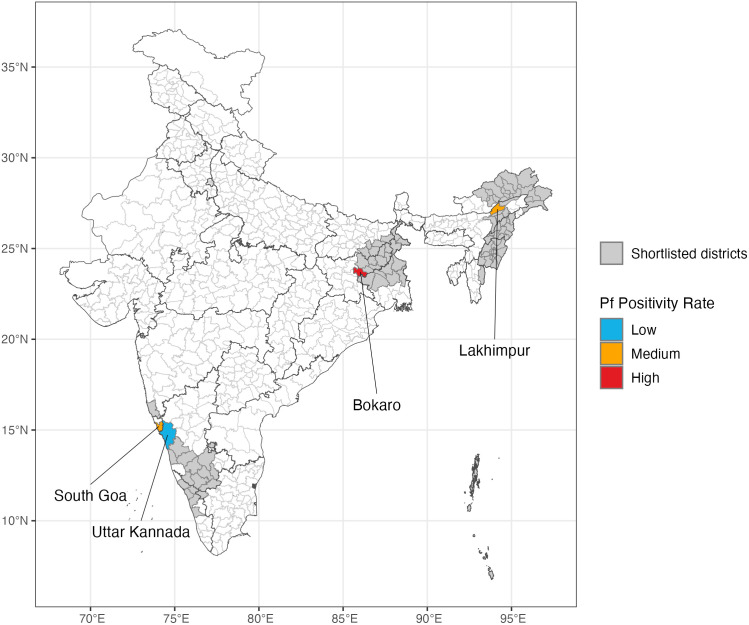
Location of 100 districts (grey) shortlisted using the model-guided decision framework, together with four districts ultimately selected, coloured by PFPR.

We selected the districts of Bokaro in Jharkhand state, Lakhimpur in Assam [31], South Goa in Goa, and Uttar Kannada in Karnataka [32] (Fig 5). These districts were selected based on available information on Pf malaria endemicity in the district or in adjacent areas, population density, and feasibility of surveys based on access, infrastructure, and local support.

## Discussion

In this paper, we describe a framework to use geostatistical modelling of existing genetic data of Pf malaria to optimise proposed surveillance of molecular markers for antimalarial resistance. Our framework is able to identify areas of the highest value to surveillance, as judged by clinical decision-makers, i.e., those areas with high predicted prevalence of molecular markers of resistance and high uncertainty in model predictions, while also incorporating other relevant epidemiological parameters. The implementation of our decision-making framework is facilitated by an RShiny dashboard that assists the systematic selection of surveillance sites to enhance surveillance coverage and increase capacity for early detection of emerging drug resistance.

The prevalence of molecular markers for SP resistance in surveys conducted from 2001 to 2022 is elevated in the Eastern and Northeastern regions of India. Molecular markers for artemisinin resistance remained mostly limited to northeastern states, with the exception of West Bengal. This was caused by a single study in which *pfk13* mutations were identified; *pfk13* mutations had not been detected in this region before and have not been detected since, despite repeated studies. The corresponding map of uncertainty in model estimates shows high uncertainty in the northeastern and southern regions of the country. This may be partly due to the binomial observation model we impose on prevalence data, for which variance is lowest at extreme prevalences (close to zero or one) and highest at prevalences close to 0.5. It is important that a decision-maker is able to review computational uncertainty, and how the uncertainty is partly shaped by the observational model imposed on prevalence data. A map of sample variance in predictions before they are rescaled to the probability scale may be another practical measure of uncertainty that is not included in our framework for brevity. It may appear paradoxical that the predicted estimates with the highest uncertainty are in regions where the most surveys have been conducted. However, this is expected given that the geospatial model takes into consideration all results collected from a location or within a region, including those from before resistance emerged in those foci. As the presence or prevalence of resistance markers is confirmed over time and with multiple surveys in agreement, the uncertainty would be expected to decrease in these regions, e.g., as markers of resistance reach saturation. The application of alternative observation models, for example the betabinomial, which may account for over dispersion in data, is the subject of future work.

The models presented here are fit to datasets including data from neighbouring countries (within 675 kilometres of Indian borders), as these can influence predictions of marker prevalence within India. This leads to some small differences between these maps and previously published predictions [16], however does not meaningfully change the results of our site selection framework

The predicted prevalence and its associated uncertainty are key determinants to identify sites or districts where performing a survey would be most valuable, i.e., those which can confirm the presence or prevalence of resistance while also reducing the practical uncertainty in future model estimates. However, these surveillance objectives must be complemented by epidemiological and demographic considerations to ensure that surveys are feasible. Here, districts were first ranked and filtered with respect to the modelled prevalence of *pfk13* mutants, then the related uncertainty, followed by ranking and filtering based on the estimated prevalence of *pfdhps* 540E. The shortlisted districts were then classified based on state-level PFPR, to ensure the complete malaria endemicity spectrum was covered in the final set of selected sites. This allowed a balance between the collection of a sufficient number of specimens from malaria patients and the coverage of areas with low endemicity where resistance may be more likely to emerge [33]. The RShiny dashboard was developed to facilitate and systematise the iterative shortlisting of sites by integrating geospatial model outputs, malaria epidemiology data and operational considerations, such as district accessibility and human population density. Finally, four districts, one each from states with high and low PFPR as well as two from states with moderate PFPR, were selected from the shortlist for this pilot exercise. This final selection was based on pragmatic considerations – population density, historical malaria case burden, and existing human resources and/or infrastructure to facilitate survey implementation. We were also able to ensure that any apparent spatial clustering in the shortlisted set of 100 districts could be accounted for in the final set of districts selected. Prospective surveillance is being conducted at each of these sites and the results of our planned surveillance study will be used for a second round of geospatial modelling along with other data published in the future.

Resistance marker prevalence data is scarce in the study area, contributing to model uncertainty. There was very limited access to the recent district-level malaria endemicity data for site filtering - this would provide more spatial precision than state-level data. There are also other factors, such as human mobility or access to treatments (i.e., selective pressure on the parasites through drug uptake), which can influence the spread or emergence of resistance but which are not yet incorporated in the geospatial model. We will be able to review the structure of our models when we begin to receive the results from initial rounds of planned surveys. However, in their current form, the models and our decision-making framework facilitate an approach to site selection for surveillance of molecular markers for antimalarial drug resistance that is systematically guided by data and modelling. The proposed framework has the potential to be adapted and implemented in other endemic regions, under local objectives and constraints.

## Conclusion

Effective disease surveillance requires optimal use of limited resources, informed by an understanding of a disease’s spatiotemporal distribution. Geospatial models are powerful tools for finding the signal in spatiotemporal data, despite the sparse and/or conflicting data collected through disease surveillance efforts. However, on their own, models do not directly allow decision-makers to evaluate alternative choices and trade-offs. The framework described in this paper provides a systematic way to incorporate geospatial modelling outputs into decision-making for the surveillance of molecular markers for antimalarial drug resistance in India, with its diverse demography, topography, and climate. The use of a dynamic dashboard created with the RShiny software package ensures decision-making is quantitatively supported by modelling outputs while keeping stakeholders informed about the current state of markers for antimalarial drug resistance. Our framework demonstrates the potential of model-guided decision-making in settings with scarce data and could be adapted for other regions or diseases. It is critical that all available data quantitatively guide decision-making if surveillance resources are to be optimally targeted.

## Supporting information

S1 Text Supplementary Methods (DOCX)
