# Supplementary material for: Model-guided geospatial surveillance system for antimalarial drug resistance
Source: PLOS Glob Public Health. 2026 Jan 6;6(1):e0004717. doi: 10.1371/journal.pgph.0004717 (PMC12773801; doi:10.1371/journal.pgph.0004717)
Supplement: S1 Text — (DOCX) [file pgph.0004717.s001.docx]

**Supplementary Methods**

The mathematical model was developed using a model-based geostatistics (MBG) framework and parameters were estimated in a Bayesian inference setting using Markov chain Monte Carlo (MCMC) simulation. The Bayesian MBG approach allows for spatial prediction within the generalised linear modelling framework as well as for parameter estimation and quantification of uncertainty.

The number of individuals in each study that were positive for a marker of interest was taken to be binomially distributed, given the number of individuals tested in the study and the probability of the marker being present. This probability was then modeled as the inverse logit transformation of the sum of a random field and an unstructured random component. The unstructured component was assumed to be independent and identically Normally distributed variables with zero mean and variance to be estimated. The random field (structured component) was modelled as a Gaussian process, with mean function that varies linearly in time and the covariance function was chosen to be a version of the spatio-temporal structure advocated by Stein (Stein, 1999) and adopted in previous work to model spatiotemporal trends in antimalarial drug resistance (Flegg et al., 2022; Flegg et al., 2024; Nain et al., 2024). The mean and covariance functions both have parameters to be estimated. Prior distributions are specified for all model parameters, see Flegg et al., 2024 for details.

The implementation of the model proceeds with two main steps: inference and prediction. In the inference stage, the output of the model was the posterior probability distribution of the model parameters given the observed data. Samples were drawn from the posterior distribution of the model mean and covariance parameters, and the random field at each location where the marker data was available, using an MCMC approach. The MCMC algorithm was implemented in the Python package PyMC (Patil et al., 2010).

In the prediction stage, the output was the posterior predictive distribution of the prevalence of the marker at each space-time point of interest; here a nominal 5 x 5 km^2^ resolution grid over India. From the output of the inference stage, parameter values were available for the sample from the posterior for each of the data locations. To generate a predictive map for a year of interest, for each of the posterior samples, for each of the prediction locations on a 5 x 5 km grid, the conditional distribution of the random field was sampled from a multivariable Normal distribution, conditional on the posterior samples. Repeating this for each of the posterior samples formed the set of marker prevalence samples for this space-time location, for which the median and standard deviation was found. Repeating for each prediction location on a 5 x 5 km grid resulted in median and standard deviation maps of marker prevalence. In this way, the uncertainties in parameters were propagated into the predictions.

**References**

Stein ML. Interpolation of spatial data: some theory for kriging. Springer Science & Business Media; 1999 Jun 22.

Spatiotemporal spread of Plasmodium falciparum mutations for resistance to sulfadoxine-pyrimethamine across Africa, 1990–2020. JA Flegg, GS Humphreys, B Montanez, T Strickland, ZJ Jacome-Meza, … PLoS computational biology 18 (8), e1010317 47 2022

Spatio-temporal spread of artemisinin resistance in Southeast Asia. JA Flegg, S Kandanaarachchi, PJ Guerin, AM Dondorp, FH Nosten, … PLoS computational biology 20 (4), e1012017

Systematic Review and Geospatial Modeling of Molecular Markers of Resistance to Artemisinins and Sulfadoxine–Pyrimethamine in Plasmodium falciparum in India. M Nain, M Dhorda, JA Flegg, A Gupta, LE Harrison, S Singh-Phulgenda, … The American journal of tropical medicine and hygiene 110 (5), 910 3 2024

Patil A, Huard D, Fonnesbeck CJ. PyMC: Bayesian Stochastic Modelling in Python. J Stat Softw. 2010 Jul;35(4):1–81.
